# The BCD of response time analysis in experimental economics

**DOI:** 10.1007/s10683-017-9528-1

**Published:** 2017-05-24

**Authors:** Leonidas Spiliopoulos, Andreas Ortmann

**Affiliations:** 1 Max Planck Institute for Human Development, Berlin, Germany; 2 University of New South Wales, Sydney, Australia

**Keywords:** Response time, Time constraints, Experimental economics, Procedural rationality, Games, Strategic decision making

## Abstract

For decisions in the wild, time is of the essence. Available decision time is often cut short through natural or artificial constraints, or is impinged upon by the opportunity cost of time. Experimental economists have only recently begun to conduct experiments with time constraints and to analyze response time (RT) data, in contrast to experimental psychologists. RT analysis has proven valuable for the identification of individual and strategic decision processes including identification of social preferences in the latter case, model comparison/selection, and the investigation of heuristics that combine speed and performance by exploiting environmental regularities. Here we focus on the benefits, challenges, and desiderata of RT analysis in strategic decision making. We argue that unlocking the potential of RT analysis requires the adoption of process-based models instead of outcome-based models, and discuss how RT in the wild can be captured by time-constrained experiments in the lab. We conclude that RT analysis holds considerable potential for experimental economics, deserves greater attention as a methodological tool, and promises important insights on strategic decision making in naturally occurring environments.

## Introduction

It seems widely agreed that decisions “in the wild” (Camerer 2000, p. 148) are often afflicted by time pressure, typically to the decision maker’s detriment. Addressing these effects of time pressure, the common adage “to sleep on it”, for example, implies that delaying a decision can improve its quality by allowing more time to reflect on it cognitively and emotionally. In fact, legislators have acknowledged the influence of the interaction of time and emotions on decisions: Mandatory “cooling-off periods” are used to temper the effects of sales tactics such as time pressure on consumer purchases by allowing consumers to renege on impulse purchases (Rekaiti and Van den Bergh 2000). Similarly, “cooling-off periods” between the filing and the issuance of a divorce decree have been found to reduce the divorce rate (Lee 2013). When time is scarce, decision makers have less time to process information pertaining to the specific case at hand, and instead may rely on their priors, which may be driven by stereotypes. Under time pressure, stereotypes about defendants are more likely to be activated and can affect judgments of guilt and proposed punishment (van Knippenberg et al. 1999). Similarly, judgments under time pressure about a suspect holding a weapon or not are more likely to exhibit racial biases (Payne 2006). Assessments of whether acute medical attention is required can also be shaped by time pressure (Thompson et al. 2008). Other examples of environments that operate under time pressure include auctions, bargaining and negotiations, urgent medical care, law enforcement, social settings with coordination issues, and human conflict; moreover, *all* decisions have an implicit non-zero opportunity cost of time. Beyond the time taken to deliberate, collecting and processing information efficiently is also time-consuming. Yet, the temporal dimension of decision making has not featured prominently in economists’ analyses of behavior. We argue below that it often matters, both for individual and strategic decision making (henceforth, individual and strategic DM). We will argue that the analysis of (possibly forced) response time (RT) data can significantly complement standard behavioral analyses of decision making; of course, it is no panacea and we will highlight challenges and pitfalls along the way.

The scientific measurement of the timing of mental processes (*mental chronometry*), starting with Donders (1868), has a long tradition in the cognitive psychology literature—see Jensen (2006), Luce (2004) and Svenson and Maule (1993) for contemporary discussions. While psychologists have long acknowledged the benefits of jointly analyzing choice and possibly forced RT data, even behavioral economists have until recently paid little attention to RT. Many of the most prominent behavioral models remain agnostic about RT, e.g., Prospect Theory (Kahneman and Tversky 1979; Tversky and Kahneman 1992), models of fairness (Bolton and Ockenfels 2000; Charness and Rabin 2002; Fehr and Schmidt 1999), and temporal discounting models (Laibson 1997).

Early work in economics can be classified into two types of RT applications. The first type of application emphasizes the usefulness of RT analysis for DM *without* any time constraints (Rubinstein 2004, 2006, 2007, 2008, 2013, 2016), which we refer to as *endogenous* RT. Decision makers are free to decide how long to deliberate on a problem; RT is shaped by the opportunity cost of time and the magnitude of the task incentives. Consequently, rational decision makers must choose a point on a speed–performance efficiency frontier. For economists, performance will typically be measured as utility. This is consistent with an unconstrained utility maximization problem only when the opportunity cost of time is very low relative to the incentives, thereby excluding a significant proportion of real-world decision environments. Researchers working with endogenous RT typically measure the time taken for a subject to reach a final (committed) decision—we refer to this as *single* RT. However, subjects’ *provisional* choices may be elicited throughout the deliberation period at various times (Agranov et al. 2015; Caplin et al. 2011)—we refer to this as *multiple* RT. Multiple RT captures the evolution of within-subject decision processes over time, yielding more useful information about the dynamic underpinnings of decision making. In most experiments, payoffs are typically independent of RT (*non-incentivized*). Another possibility is to use *incentivized* tasks that introduce a benefit to answering more quickly, for example, by having a time-based payoff reward or penalty (e.g., Kocher and Sutter 2006).^1^

The second type of application emphasizes the examination of DM under * time constraints* (Kocher et al. 2013; Kocher and Sutter 2006; Sutter et al. 2003), which we refer to as* exogenous* RT. The most common type of time constraint is *time pressure*, i.e., limited time to make a decision. *Time delay*, i.e., the imposition of a minimum amount of time, can be also found in some studies, usually those interested in the effects of emotions on decision making (e.g., Grimm and Mengel 2011). Decision makers are increasingly being called upon to multi-task, i.e., handle many tasks and decisions almost simultaneously or handle a fixed number of tasks within a time constraint. Measuring the time allocated to individual tasks is crucial to understanding how decision makers prioritize and allocate their attention. One technique of implementing time pressure in the lab is to impose a time limit *per set* of tasks, instead of *per task*, as is typically done. This route is taken by Gabaix et al. (2006), who find qualitative evidence of efficient time allocation, i.e., subjects allocated more time to tasks that were more difficult. In the majority of studies, treatments within an experiment typically compare an endogenous RT treatment with other exogenous RT constraints, i.e., RT is the *only* variable that is manipulated across treatments. However, if other variables are also simultaneously manipulated across treatments, it is possible that the RT manipulations will interact to different degrees with the other variables. Furthermore, knowledge that an opponent is also under time pressure could induce a change in beliefs about how the opponent will behave. These two examples highlight the importance of a thorough understanding of RT constraints and a well-designed experiment that minimizes the impact of such issues—we return to the issue of identification later in Sect. 5.1.

Endogenous and exogenous RT analyses differ in the benefits that they offer. The former’s usefulness lies primarily in revealing additional information about a decision maker’s underlying cognitive processes or preferences (aiding in the classification of decision-maker types) and the effects of deliberation costs on behavior. The latter’s usefulness lies primarily in exploring the robustness of existing models to different time constraints, i.e., verifying the external validity of models and the degree to which they generalize effectively to different temporal environments. We will present evidence that behavior on balance is strongly conditional on the time available to make a decision. In fact even the *perception* of time pressure, when none exists, can significantly affect behavior (Benson 1993; DeDonno and Demaree 2008; Maule et al. 2000).

Experimental designs manipulating realistic time constraints in the lab are a useful tool to advance our understanding of behavior and adaptation to time constraints. Exogenous RT analysis has already led to important insights within the context of two different approaches to modeling decision making. The first approach examines how *decision processes* change under time pressure. Historically, this has been the focus of research in cognitive psychology that was driven by the belief that cognition and decision making rules are shaped by the environment (Gigerenzer 1988; Gigerenzer et al. 1999, 2011; Gigerenzer and Selten 2002; Hogarth and Karelaia 2005, 2006, 2007; Karelaia and Hogarth 2008; Payne et al. 1988, 1993). By exploiting statistical characteristics of the environment, such ecologically rational heuristics (or decision rules) are particularly robust, even outperforming more complex decision rules in niche environments. This raises the following question. How are the appropriate heuristics chosen for environments with different temporal characteristics? A consensus has emerged from this literature that time pressure leads to systematic shifts in information search and, ultimately, selected decision rules (Payne et al. 1988, 1996; Rieskamp and Hoffrage 2008). Subjects adapt to time pressure by: (a) acquiring less information, (b) accelerating information acquisition, and (c) shifting from alternative—towards attribute-based processing, i.e., towards a selective evaluation of a subset of the choice characteristics. These insights from cognitive psychology emerged from individual DM; in Spiliopoulos et al. (2015) we present evidence that similar insights can be had for strategic DM. Imposing time pressure in one-shot $3\times 3$ normal-form games led to changes in information search (both acceleration and more selective information acquisition) that also have been documented for individual DM as well as the increased use of simpler decision rules such as Level-1 reasoning (Costa-Gomes et al. 2001; Stahl and Wilson 1995).^2^

The second approach examines how *preferences* may depend on time constraints. This approach contributes to the discussion on the (in)stability or context-dependence of preferences by adding the temporal dimension to the debate (Friedman et al. 2014). Specifically, we will review evidence that a wide range of preferences are moderated by time constraints. For example, risk preferences are affected by time pressure. Risk seeking (or gain seeking relative to an aspiration level) can be stronger under time pressure in the gain or mixed domains, although this may depend on framing (e.g., Kocher et al. 2013; Saqib and Chan 2015; Young et al. 2012). Furthermore, RT analysis has led to a burgeoning inter-disciplinary literature and debate about the relationship between social preferences and RT (both endogenous and exogenous). A debate is in progress about whether pro-social behavior is intuitive, and whether people are more likely to behave more selfishly under time pressure (e.g., Piovesan and Wengström 2009; Rand et al. 2012, 2014; Tinghög et al. 2013; Verkoeijen and Bouwmeester 2014). This is one of the most exciting topics that RT analysis has motivated, as the nature of human cooperation is central to our understanding of the functioning of society—we will discuss this debate in detail in the next section.

The analysis of endogenous RT–while not as common–has also produced some interesting findings in experimental economics. Recall, that endogenous RT analysis is primarily a methodological tool that allows researchers to learn more about individuals’ decision processes and preferences, which tend to be quite heterogeneous. Consequently, researchers are often interested in the classification of decision-makers into a set of types based, say, on social preferences and risk preferences. Classification is typically accomplished solely on the basis of choices (through revealed preferences), but response time can also be used for this purpose. Numerous studies have determined that RT can be used to predict behavior out-of-sample or to classify subjects into types, often more efficiently than using other classical variables such as the level of risk aversion (Rubinstein 2013) and even normative solutions (Schotter and Trevino 2014b). Chabris et al. (2008, 2009) found that intertemporal discount parameters estimated using only RT data were almost as predictive as those estimated traditionally from choice data. Rubinstein (2016) proposes classifying players within a spectrum called the contemplative index. The degree of contemplation or deliberation that a person exhibits seems to be a relatively stable personality trait, which can be used to predict behavior even across games.

While experimental economists have begun tapping into the potential of exogenous RT analysis, they have not embraced endogenous RT analysis to the same degree. It is our belief that there still exists significant potential for the latter; however, similarly to the endogenous RT work in cognitive psychology, unleashing the full potential is aided by the use of procedural (process-based), rather than substantive (outcome-based), models of behavior. In contrast to substantive models, procedural models stipulate *how* decisions are made (specifying the mechanisms and processes) in addition to the resulting decisions. Procedural models that jointly predict choice and RT are crucial for predicting how adaptation occurs in response to RT constraints—the class of sequential-sampling models discussed in Sect. 3.6 is one example. In mathematical psychology, model comparisons of procedural models have a tradition of using RT predictions (not just choices) to falsify models—see for example Marewski and Melhorn (2011). Our literature review^3^ revealed that the existing RT studies in economics exhibit a lack of formal procedural modeling and are most often viewed through the lens of dual-system models (Kahneman 2011). These models contrast a faster, more instinctive and emotional System 1 with a slower, more deliberative System 2—under time pressure System 1 is more likely to be activated. Many studies on social preferences are devoted to reverse inference based on these two systems, i.e., types of decisions that are made faster are categorized as intuitive. This may be a problematic identification strategy.

We have briefly presented what we see as examples of how RT analysis has already led to important insights in experimental economics. The case for collecting RT data in economic experiments seems strong, as RT is an additional variable available at virtually zero cost for all computerized experiments. If no time constraints are imposed, the collection of RT data is not noticeable by experimental subjects and neither primes nor otherwise affects their behavior. While there is little cost associated with collecting the data, the benefits depend on the type of study. Response time analysis seems particularly useful in the cases that we have outlined above where time constraints may mediate decision-makers’ preferences (e.g., risk or social preferences) or processes. Also, in information-rich environments where information search or retrieval may be costly, the imposition of a time constraint or high opportunity cost of time is likely to have an amplified effect on behavior. In empirical model comparison studies,^4^ where it is practically difficult to collect enough choice data on a large enough set of tasks, RT can be used to more effectively discriminate between procedural models by increasing the falsifiability of models (they may be rejected either for poor choice predictions or poor RT predictions). Finally, even basic response time analysis can be useful in virtually any experimental study. Extremely quick responses or very slow responses are often symptomatic of subjects that are not engaging with the experiment seriously. The influence of such outliers on the conclusions drawn from experiments can be extremely problematic as we will show later on.

Our manuscript is meant to assess the state of the art, to stimulate the discussion on RT analysis, and to bring about a critical re-evaluation of the relevance of the temporal dimension in decision making. In complex strategic decision making, adaptive behavior that makes efficient use of less information, less complex calculations (e.g., such as higher-order belief formation about an opponent’s play), and emergent social norms, seems even more important than for individual DM (Hertwig et al. 2013). Inspired by the results in cognitive psychology, we envision a research agenda for strategic DM that parallels that of individual DM. Whilst we emphasize the potential contribution of RT to strategic DM, we note that most of our arguments are relevant to individual DM.

We envision this manuscript as a critical review of RT analysis that is accessible to readers with little prior knowledge of the topic, for instance an advanced graduate student who wants to jump-start her/his understanding of the issue. Since the paper is quite long, we have used a modular structure so that readers with prior experience may selectively choose the sub-topics they are more interested in. An extended version of the paper including some more technical arguments can be found in our working paper (Spiliopoulos and Ortmann 2016), which we first posted in 2013 and have revised contemporaneously.

The present manuscript is organized as follows. We summarize the benefits, challenges, and desiderata (the BCD) of both the experimental implementation of RT experiments and the joint statistical modeling of choice and RT data in Table 1. A literature review of RT studies and summary of the most important findings follows in Sect. 2. In the following section we delve into the multitude of ways to model RT and choices (Sect. 3). We then devote the next three sections to pull together the benefits, challenges, and desiderata of RT analysis in experimental economics. We encourage the reader to preview our summary arguments in Table 1—keeping these arguments in mind before delving into detailed arguments will likely be beneficial. Section 7 concludes our manuscript. A detailed literature review of RT in strategic DM is presented in “Appendix 1”, including Table 3 taxonomizing all the studies we have found. A framework for relating behavior and decision processes to time constraints for strategic DM is presented in “Appendix 2”.

**Table 1:** Response time analysis: benefits, challenges, and desiderata

	Sect.	p.	Summary
Benefits			
Improved external validity	4.1	14	Decisions in the wild are often made under time constraints and influenced by the opportunity cost of time
Mapping the relationship between RT and performance	4.2	14	Decision makers may tailor the balance between speed and performance to the environment and to their own goals and constraints
Explicit experimental control of RT	4.3	15	Experiments without explicit time constraints may have ambiguous implicit constraints
Improved model selection, identification and parameter estimation	4.4	16	RT data provide further information about the underlying decision processes. Joint estimation of both choice and RT data improves the precision of parameter estimates in behavioral models
Classification of heterogeneous types	4.5	16	RT data can be used to classify heterogeneous subjects into more finely delineated types
RT as a proxy for other variables	4.6	16	RT can be useful as a proxy for unobserved effort and/or strength of preference
Challenges			
Identification	5.1	16	Due to the multitude and possible combinations of decision processes, identification of procedural models is challenging even with the addition of RT data
Irregular RT distributions, outliers and non-responses	5.2	17	The selection of analytical methods requires caution analysis as RT distributions tend to be non-normal. Outliers are very common and how they are handled can significantly affect conclusions
Heterogeneity	5.3	17	Between-subjects heterogeneity may be very high for strategic DM, which admits a large number of different beliefs and strategies
RT measurement error	5.4	17	Differences in software, hardware or network latency can lead to measurement errors
Desiderata			
Procedural modeling	6.1	18	Procedural models that make falsifiable predictions about the joint distribution of choice/RT are preferable
Concurrent collection of other process variables	6.2	18	The potential benefits of RT data are maximized when coupled with other process variables, such as elicited beliefs, information search, et cetera
Hierarchical latent-class modeling	6.3	19	Effectively captures heterogeneity in behavioral models and their parameters and deals with the problem of outliers
Cross-validation	6.4	19	Aids in model comparison and prevents overfitting by overly complex models. Behavioral models should be able to predict choice from RT and vice-versa
Experimental implementation	6.5	19	Factorial designs varying the degree of time pressure and task difficulty are effective in identifying decision processes

## A review of the RT literature

There are three waves of RT studies that can be classified according to the types of tasks investigated. The first wave was concerned with judgment tasks, for example involving perceptual acuity or memory retrieval. A second wave emerged first in cognitive psychology and later in economics examining individual DM choice tasks that required valuation processing rather than judgments, e.g., decision making under risk and lottery choices (Dror et al. 1999; Kocher et al. 2013; Wilcox 1993), and multi-alternative and -attribute choice (Rieskamp and Hoffrage 2008; Rieskamp and Otto 2006). The third, and most recent, wave involves the analysis of RT in strategic DM or games—below we focus on this third wave.

We catalogued the existing literature on RT in strategic games by performing multiple searches in Google Scholar (April, 2013)^5^ and by sifting through the results of these searches to obtain an initial list of relevant studies. We then identified other studies on RT that were cited in these papers to obtain as complete a list as possible. We have repeated these searches for each revised draft since the original. Unpublished working papers are included because RT studies in strategic DM emerged fairly recently.

A summary of the main characteristics of the literature using RT in strategic DM can be found in our working paper (Spiliopoulos and Ortmann 2016). A more detailed discussion of individual studies is presented in Table 3 in “Appendix 1”. Out of a total of 52 studies (41 published and 11 unpublished) roughly half of the studies (52%) in our data set do not impose any time constraints and simply measure the (endogenous) RT of decisions. Dual-system models of behavior are the most common (48%), followed by models involving iterated strategic reasoning (15%), and models based on the cost and benefits of cognitive effort (12%) and the effect of emotions (13%).

We proceed below by discussing the key findings of the literature for the following themes broached in the introduction: (a) preferences (risk, intertemporal and social), (b) decision processes and emotions, (c) type classification, and (d) the speed–performance profile. Table 2 summarizes the key findings in each of these topics.

**Table 2:** A summary of current findings in the literature

	Main findings
Preferences	
Risk	Evidence that time pressure increases risk taking behavior in the domain of gains, but decreases risk taking behavior in the domain of losses. Framing has been found to mediate these effects, with aspiration levels playing a role.
Intertemporal	Limited evidence that the present-bias is reduced under time pressure, but the long-term discounting factor and utility function curvature remain the same.
Social	No consensus on whether cooperation or pro-social behavior are more intuitive. The debate now centers on methodological critiques based on important mediators and/or confounding variables. An alternative hypothesis with some empirical support is that reciprocity is more intuitive. Another hypothesis is that the higher the cognitive dissonance or conflict the slower the RT—this is consistent with a sequential-sampling account. This implies an inverted-U shaped relationship between RT and cooperation, which could reconcile the conflicting findings in the literature.
Processes	The closer the valuations of competing options are, the longer the (endogenous) time taken to decide. Limited evidence that the existence of aspiration levels that easily discriminate between options leads to a shorter endogenous RT.
	Decisions consistent with focal outcomes are associated with shorter RT.
Heuristics	Heuristics are more likely to be used under time pressure–in many cases they involve ignoring some of the available information, particularly in strategic DM.
Emotions	Limited evidence that time delays reduce negative emotions about unfair offers, leading to greater acceptance rates in ultimatum games.
Classification	RT is predictive of behavior (out-of-sample) in a variety of tasks. In many cases, RT is more informative that other variables such as risk preferences or the normative equilibrium solution.
Speed–performance profile	Moderate evidence that, on average, decision quality and payoff performance for individual DM is reduced under time pressure and that there exists a positive relationship between endogenous RT and performance. However, this finding is not robust for strategic DM as it depends crucially on the characteristics of a game. Preliminary findings that time-based incentives do not affect decision quality.

### Preferences and RT

#### Risk preferences

With the exception of an early study using mixed prospects (Ben Zur and Breznitz 1981), the majority of studies find that time pressure tends to increase risk-seeking behavior in the gain domain. Modeling choices between binary lotteries in a Prospect Theory framework, Young et al. (2012) find evidence of increased risk-seeking for gains under time pressure. Similarly, Saqib and Chan (2015) show that time pressure can lead to a reversal of the typical CPT preferences, so that decision makers are risk-seeking over gains and risk-averse over losses. Dror et al. (1999) find that time pressure in a modified blackjack task induced a polarization effect—participants were more conservative for low levels of risk but were more risk-seeking for high levels of risk. Financial decision making, particularly trading, is often performed on a much fast time scale of the order of a few seconds. Nursimulu and Bossaerts (2014) discover that under time pressure, subjects were risk-averse for a 1 s (one second) time constraint but risk-neutral for 3 and 5 s constraints, and positive skewness-averse for a 1 s constraint with their aversion increasing in the 3 and 5 s constraints. Kocher et al. (2013) tell a more cautionary tale about the robustness of time pressure effects on risk attitudes. They conclude that (a) risk seeking in the loss domain changes to risk aversion under time pressure, (b) risk aversion is consistently found with, and without, time pressure in the gain domain, and (c) for mixed lotteries, conditional on the framing of the prospects, subjects can become more loss-averse but also exhibit gain-seeking behavior. These studies involved decisions from description rather than experience.^6^ Madan et al. (2015) confirm that time pressure in decisions from experience also leads to an increase in risk-seeking behavior.

The evidence that time constraints moderate risk preferences is an important one for real-world decision making. Many high-stakes financial and medical decisions are made under time pressure—if decision makers exhibit more risk-seeking at the time of decision, then this could leave them open to the possibility of larger losses than their (non time-constrained) preferences would dictate after the decision is made.

#### Social preferences

Tasks involving social preferences dominate the strategic DM literature—ultimatum, public goods and dictator games comprise approximately one-quarter, one-fifth, and one-tenth of the studies respectively. We taxonomize the literature according to numerous hypotheses regarding the relationship between RT and behavior. The *costly-information-search* hypothesis (our own term) claims that response time is positively correlated with pro-social behavior because it requires attending to more information (the payoffs of the other player) and thinking about how to trade-off the various payoff allocations among players. In this tradition, Fischbacher et al. (2013) study mini-ultimatum games and find evidence that RT is increasing in the social properties subjects lexicographically search for, such as fairness, kindness and reciprocity. On the other hand, the *social-heuristics* hypothesis (Rand et al. 2014)—sometimes more broadly construed as the *fairness-is-intuitive* hypothesis–contends that pro-social behavior is an intuitive or instinctive response in humans, suggesting a negative relationship between RT and pro-social behavior.

The social-heuristics hypothesis is the most tested in the literature as it is compatible with popular dual-system explanations of behavior, which use RT to infer what types of responses are instinctive or deliberative. Rand et al. (2012, 2014) find that cooperation is more intuitive than self-interested behavior, as they find a negative relationship between cooperation and both endogenous RT and time pressure. Supporting the hypothesis that cooperative behavior is instinctive, Lotito et al. (2013) conclude that contributions and RT are negatively related in public good games. Furthermore, focusing on responder behavior in the ultimatum game, Halali et al. (2011) find that subjects reject an unfair offer more quickly than they accept it. In dictator games, Cappelen et al. (2016) also conclude that fair behavior is faster.

However, other studies contest this hypothesis on various grounds, primarily methodological. Tinghög et al. (2013) disagree with Rand et al. (2012) on the basis that including some RT outliers in the data, leads to the conclusion that there is no clear relationship between RT and the degree of cooperation. In a public goods game, Verkoeijen and Bouwmeester (2014) manipulate knowledge about other players’ contributions, the identity of an opponent (human or computer) under both time pressure and time delay; they do not find a consistent effect of time constraints on the degree of cooperation. In ultimatum games under time pressure, Cappelletti et al. (2011) find that proposers make higher offers whereas Sutter et al. (2003) find that responders are more likely to reject offers. In dictator games, Piovesan and Wengström (2009) conclude that RT is shorter for selfish choices both within- and between-subjects.

One of the most popular alternative hypotheses suggests that RT is increasing in the degree of cognitive dissonance or conflict that a decision maker is facing. Matthey and Regner (2011) induced cognitive dissonance in subjects by allowing them to decide whether they wish to learn about their opponents’ payoff function. They discovered that the majority of otherwise “pro-social” subjects prefer not to view their opponents’ payoff (when possible) using their ignorance as an excuse to act selfishly without harming their self-image. Choosing to ignore information, however, by inducing cognitive dissonance led to shorter RTs. In line with Dana et al. (2007), they conclude that many subjects are mainly interested in managing their self-image or others’ perception rather than being pro-social. Jiang (2013) finds that honest choices in cheating games were associated with longer RT, suggesting again that people experience cognitive dissonance or must exert self-control to choose a non-selfish action. Evans et al. (2015) argue that the disparate findings concerning the relationship between cooperation and RT can be reconciled under the assumption that greater decision conflict is associated with longer RTs. Consequently, non-conflicted (extreme) decisions, such as purely selfish or purely cooperative behavior, will typically be faster than conflicted decisions attempting to reconcile both types of incentives. This leads to an inverse-U shaped relationship between RT and cooperation rather than the linear relationship typically postulated in the literature. In a meta-analysis of repeated games, Nishi et al. (2016) conclude that RT is driven not by the distinction between cooperation and self-interest, but instead by the distinction between reciprocal and non-reciprocal behavior. In social environments that are cooperative, cooperation is faster than defection, but in non-cooperative environments the reverse holds. The authors put forth an explanation based on cognitive conflict, i.e., non-reciprocal behavior induces cognitive conflict in the decision-maker. Finally, Dyrkacz and Krawczyk (2015) argue that subjects in dictator and other games are more averse to inequality under time pressure.

Another explanation focuses on the possibility that imposing time pressure has unwanted side-effects, in particular it might create confusion about the game leading to more errors. Inference about social preferences can be problematic if these errors are systematically correlated with RT. In a repeated public-goods game, Recalde et al. (2015) find that the shorter RT is, the more likely errors are. Ignoring this relationship would lead researchers to conclude that subjects with shorter RTs had stronger pro-social preferences. Goeschl et al. (2016) also confirm that some subjects are confused in public goods games, and find a heterogeneous effect of time pressure on players. Subjects who were clearly not confused about the game became more selfish under time pressure.

On an important methodological note, there may exist other mediators of RT–that likely differ across studies–which must be rigorously accounted for before inference can be made. Krajbich et al. (2015) critique the use of RT to infer whether strategies are instinctive or deliberative without explicitly accounting for task difficulty and the heterogeneity in subject types, i.e., what is intuitive for each individual may depend on their type. Along these lines, Merkel and Lohse (2016) do not find evidence for the “fairness is intuitive” hypothesis after controlling for the subjective difficulty of making a choice. Similarly Myrseth and Wollbrant (2016), in a commentary on Cappelen et al. (2016), also draw attention to the importance of other similar mediators, making reverse-inference problematic, i.e., inferring that faster decisions are more intuitive. They make an important argument regarding the validity of drawing conclusions from absolute versus relative response times. Faster response times in various treatments may still be slow enough to reasonably lie in the domain of deliberate decision processes.

In light of the above studies and the methodological critiques that have been voiced, we believe that firm conclusions should not be drawn yet regarding the relationship between social preferences and RTs. While individual studies often test one or two of these competing hypotheses, nothing precludes the relevance of many hypotheses especially when possible mediators are concerned. For example, assume that pro-social behavior is the more intuitive response. However, if making the pro-social decision involves significant information search costs (about the opponent’s payoffs), then it is possible for the *total* RT to still be longer for pro-social behavior—this depends on the proportion of total RT that is spent on information search. Consequently, accounting for different sub-processes of decision making and the time required to execute these sub-processes could be important (a more extensive discussion of this can be found in “Appendix 2”). Future studies should aim at controlling rigorously for the possible mediators that have been brought up and competitively testing the various hypothesis within the same framework.

#### Intertemporal preferences

Lindner and Rose (2016) conclude that while long-run discounting and utility function curvature are quite stable, present-biased preferences are significantly reduced under time pressure. They attribute this finding to a change in the attention of subjects, who were found to focus relatively more on the magnitude, rather than the timing, of payoffs. This is a striking result, as a dual-system account would predict that under time pressure, System I will be activated, leading to more impulsive choices, i.e., an increase in present bias. Again, we note that changes in attention and information search must be examined before reaching conclusions. The lack of studies examining intertemporal preferences and time is notable–further work is necessary to draw robust conclusions.

### Decision processes and RT

Sequential-sampling models of decision making (also referred to as information-accumulation, or drift-diffusion models) have become one of the main paradigms in the mathematical/cognitive psychology literature (Busemeyer 2002; Ratcliff and Smith 2004; Smith 2000; Smith and Ratcliff 2004; Usher and McClelland 2001)—see also the extensive discussion in Sect. 3.6. These models assume that cognitive systems are inherently noisy and that the process of arriving at a decision occurs through the accumulation (integration) of noisy samples of evidence until a decision threshold is reached. An important prediction of these models is that the smaller the difference in the values of the options, the longer the RT. Krajbich et al. (2012)—see also similar work in Krajbich et al. (2010) and Krajbich and Rangel (2011)—extend standard sequential-sampling models to explicitly incorporate the allocation of attention and show that their model can simultaneously account for the triptych of information lookups, choice and RT. Importantly, their model predicts that the time spent on information lookups can influence choice, and that time pressure can lead to noisier valuations, thereby increasing the probability of an error.

Similar conclusions have been reached in the economics literature, albeit derived from different models. Wilcox (1993) finds that subjects exhibit longer RT–a proxy for effort–in a lottery choice task when monetary incentives are higher and the task is complex. Gabaix and Laibson (2005) and Gabaix et al. (2006) also derived the above-mentioned relationship between RT and the difference between option valuations under the assumption that valuations are noisy, but improve the more time is devoted to the task—more details on their modeling can be found in Sect. 3.5. Chabris et al. (2009) tested the optimal allocation of time in decision tasks and reported empirical evidence that the closer the expected utility of the competing options is, the longer the response time. Similarly, Chabris et al. (2008) find that the same principle can be used to recover discount rates from RT data without observing choices.

Another important theme in the literature is the explicit consideration of heuristics (including the use of focal points) versus compensatory, and more complex, decision rules. Guida and Devetag (2013) combine eye-tracking and RT analysis in normal-form games, and find that RT was shorter for games with a clear focal point, and longer for Nash equilibrium choices. Fischbacher et al. (2013) find that participants’ behavior, although heterogeneous, is consistent with the sequential application of three motives in lexicographic fashion. The more motives that are considered, the longer the RT, e.g., a selfish type only examines own payoffs, whereas a pro-social type must also examine others’ payoffs. Coricelli et al. (2016), on the other hand, argue that choices between lotteries–whenever possible–may be driven by a simplifying heuristic based on aspiration levels. Such an aspiration-based heuristic can be executed more quickly than the compensatory processes that subjects revert to when this heuristic is not applicable. Spiliopoulos et al. (2015) found that subjects under time pressure shifted to simpler–yet still effective–heuristics, namely the Level-1 heuristic that simply best responds to the belief that an opponent randomizes with equal probability over his/her action space. Spiliopoulos (2016) examines repeated constant-sum games and finds that RT is dependent on the interaction of two different decision rules: the win-stay/lose-shift heuristic and a more complex pattern-detecting reinforcement learning model. While the former is executed faster than the latter, response time was longer when the two decision rules gave conflicting recommendations regarding which action to choose in the next round.

Research on the impact of emotions is less common. Grimm and Mengel (2011) delay participants’ decisions whether to accept/reject an offer in an Ultimatum game for ten minutes. In line with their hypothesis that negative emotions are attenuated as time passes, they find higher acceptance rates after the time delay. Although regret and disappointment have been found to play a role in choices under risk (e.g., Bault et al. 2016; Coricelli et al. 2005; Coricelli and Rustichini 2009), their relationship with RT has not been thoroughly investigated.

### Classification

RT is also used to classify subjects into different types, above and beyond possible classifications according to choice behavior. For example, Rubinstein (2007, 2013) show that a typology based on RT is more predictive than a typology based on the estimated level of risk aversion. Rubinstein (2016) objectively defines contemplative (instinctive) actions in ten different games as those actions with longer (shorter) RT than the average RT in the game for all actions. The contemplative index of a player derived from subsets of nine of the ten games was positively correlated to the probability of the same player choosing a contemplative action in the tenth game.

Devetag et al. (2016) find that the time spent looking up each payoff in $3\times 3$ normal form games is predictive of final choices and the level of strategic reasoning of players. Schotter and Trevino (2014b) use RT in global games to distinguish between two types of players with respect to their learning process. *Intuitionists* who have a eureka moment when they realize which strategy is effective and *learners* who acquire an effective strategy through a slower trial-and-error (or inductive) process. A striking result is that RT was more predictive of out-of-sample behavior than the equilibrium solution.

These findings show that RT can be used either alone or in conjunction with choice data to sharpen the classification of subjects into types, thereby increasing our ability to predict the behavior of decision makers across different tasks. This suggests that models including both choice and RT predictions have greater scope and are more generalizable to new situations (Busemeyer and Wang 2000), thereby increasing the predictive content of behavioral models.

### Speed–performance profile

Another theme in the literature relates time pressure and the opportunity cost of RT to the quality of decision making, i.e., the speed–performance relationship (discussed at length in Sect. 4.2). Kocher and Sutter (2006) found that time pressure reduced the quality of individual DM, but time-based incentives led to faster decisions without a decrease in decision quality. Arad and Rubinstein (2012) discover that higher average payoffs are achieved by subjects with longer (endogenous) RT. We believe that this theme, which is closely related to the adaptive decision maker hypothesis is the least studied so far in strategic DM. The allocation of time between a set of tasks has been studied by Chabris et al. (2009). Subjects allocated more time to those tasks that were more difficult, defined as tasks where alternative options had more similar valuations. Recall that Spiliopoulos et al. (2015) find that roughly one-third of subjects adapt strategically to time pressure without sacrificing performance (here, payoffs) despite switching to less sophisticated heuristics. There is much work to be done in understanding the speed–performance relationship in strategic DM, and examining whether it is robust to context and tasks. We conjecture it is not, therefore further work will be required to map out how and why this relationship changes—we return to this in more detail in Sect. 4.2.

### Summary

Our review of the existing literature revealed significant evidence that RT matters in decision making. Decision makers typically adapt to time constraints leading to significantly different behavior. Consequently, the generalizability of empirical findings from the lab and the scope of existing models of behavior may need to be revised. Future work should be directed toward rigorously testing the robustness of some of the main findings in experimental economics and enriching our models with procedural components that can predict how decision makers adapt to the temporal aspect of decision environments—the following section is devoted to the latter.

## Methodology—modeling

Studies of RT fall into two main categories based on how they utilize RT data, i.e., the type of model they employ. The non-procedural (descriptive) approach simply uses RT data as a diagnostic tool, thereby not requiring the specification of a model of RT per se. Consequently, the informativeness of such an approach is restricted to comparing RT across treatments. This approach can still inform us about the appropriateness of a model, the existence (or not) of significant heterogeneity in subjects’ behavior and ultimately add another criterion upon which to base classification of subjects into types. A prime example is the dual-system approach, where RT is used to classify actions/behavior as instinctive or deliberative. As of now, the majority of strategic DM studies in the literature have adopted this type of analysis. Procedural models are more falsifiable though: in addition to choice predictions they also make RT predictions, thereby sharpening model selection and comparison—see Sect. 4.4 for more details. The reader ought to relate the following discussion back to Table 4 to fully understand which processes and types of adaptation these competing models can capture.

### Dual-system models

Dual-system (or dual-process) theories, based on the assumption that the human brain is figuratively comprised of two different systems, are increasingly being applied to decision making (Kahneman 2011). For an overview of the implications of dual-system models for economic behavior, see also Alós-Ferrer and Strack (2014) and other articles in the special issue on dual-system models in the *Journal of Economic Psychology* of which it was a part. System 1, the intuitive system, is conceptualized as being influenced by emotions, instinct and/or simple cognitive computations occurring below the level of consciousness. Decisions are made quickly and do not require vast reams of information. This system is viewed as part of the earlier evolution of the human brain and tends to be associated with “older” areas of the brain, e.g., the fight-or-flight system. System 2, the deliberative system, is conceptualized to operate on the conscious level and involves higher-level cognitive processes. Decisions are made more slowly and can involve conscious information search. This system is viewed as a more recent evolution of the human brain and its usefulness involves the ability to override the instinctual responses of System 1 when necessary, or to plan a cognitive response in a new environment. Although there is evidence of some degree of localization of these systems, the double-dissociation studies often presented as evidence of two literally distinct systems at the neural level is not without controversy—see Keren and Schul (2009), Rustichini (2008) and Ortmann (2008) for critiques and comparisons of unitary versus dual system models.

We consider standard dual-system models to be primarily descriptive models of behavior rather than procedural models. We base this assessment on how dual-system models are applied rather than their potential. Typically they are used to classify behavior as instinctive or deliberative. The inherent freedom in classifying behaviors as instinctive or deliberative is an important issue with the dual-system approach, particularly for strategic DM. Rubinstein (2007) uses the following possible classifications for an instinctive response, depending on the strategic structure of the game.

1. The number of iterations required to reach the subgame perfect NE.
2. The strategy associated with the highest own payoff.
3. The number of steps of iterated dominance required to solve a game.
4. The strategy selected by self-interested individuals.
5. The strategy that yields a “fair” outcome.

There are other criteria that could define an instinctive response. In one-shot games, Guida and Devetag (2013) find that RT is smaller for games with a focal point compared to those without. In sum, definitions of instinctive responses can be very task- and context-dependent. The contradictory findings for games where social preferences are dominant provide striking evidence of this. Some studies conclude that RT is lower for self-interested choices (Brañas-Garza et al. 2016; Fischbacher et al. 2013; Matthey and Regner 2011; Piovesan and Wengström 2009), whereas other studies find that the equitable or “fair” split is associated with a lower RT (Cappelletti et al. 2011; Halali et al. 2011; Lotito et al. 2013). Under the auxiliary assumption that instinctive choices require less time, these studies arrive at opposing conclusions of what behavior has evolved to be instinctive. Furthermore, as already briefly indicated, the use of reverse inference–observing which choices are faster and declaring them to be intuitive–has been contested (Krajbich et al. 2015). The basic idea of these critical authors makes use of people’s well-documented heterogeneity, for example in social preferences, and they propose essentially that one’s basic disposition (being selfish, or being altruistic for example), determines what one considers intuitive. An alternative to the instinctive versus deliberative dichotomy, relates the computational complexity of different (procedural) decision rules to endogenous or exogenous RT (Spiliopoulos et al. 2015).

Extending the currently primarily descriptive models to include procedural sub-models for each system, and an explicit mechanism for how the two systems interact, would transform them into procedural models. Since System 2 can override System 1, a complete theory would require a specification of how, and when, this occurs. Empirical findings suggest that System 2 is less likely to control the decision if there is time pressure, cognitive load, scarcity of information, etc. (Kahneman 2003). However, the multitude of switching mechanisms currently proposed combined with the dual systems, which individually can account for different behavior, leads to the possibility of ad-hoc explanations of empirical findings.

A new generation of dual-system type models address these concerns by explicitly modeling the interaction of the systems. Models of dual selves do so by explicitly defining the role of each self and imposing structure on their strategic interactions (Fudenberg and Levine 2006, 2012; Fudenberg et al. 2014). The long-run self cares about future payoffs, whereas the short-run self cares only about short-run, typically immediate, payoffs. The short-run self is in control of the final decision made. The long-run self seeks to influence the utility function of the short-run self, but incurs a self-control cost. Such an explicit representation of the dual selves and their interaction permits sharper predictions of behavior than standard dual-system models. While these models do not explicitly account for time, it is possible to operationalize RT with the auxiliary assumption that it is increasing in the cost function of self-control. Achtziger and Alós-Ferrer (2014) and Alós-Ferrer (2016) propose a dual-process model in which the interaction between a faster, automatic process and a slower, controlled process is explicitly defined. The model’s RT predictions, for both erroneous and correct decisions conditional on the degree of conflict or agreement of the two processes, were empirically verified in a belief-updating experiment. Spiliopoulos (2016) similarly validates the model’s qualitative RT predictions in repeated games, where the automatic process is specified as the win-stay/lose-shift heuristic and the controlled process as the pattern-detecting reinforcement learning model introduced in Spiliopoulos (2013). Conflict between the two processes led to longer RT, and also influenced RTs conditional on the interaction between conflict and which process the chosen action was consistent with.

### Heuristics and the adaptive toolbox

Heuristics–often referred to as fast and frugal–in the tradition of the ecological-rationality program (Gigerenzer et al. 1999; Ortmann and Spiliopoulos 2017), are simple decision rules that often perform as well, if not better, than more complex decision rules for out-of-sample predictions, i.e., cross-validation. Heuristics are particularly amenable to response time analyses because their sub-processes and interactions are typically explicitly specified in the definition of the heuristic.^7^ Consequently, RT can be defined as an increasing function of the number of elementary information processing (EIP) units required to execute a decision rule (Payne et al. 1992, 1993). EIPs can be thought of as the lowest level operations required for the execution of a computational algorithm. These would include retrieving units of information, and processing them, e.g, mathematical operations such as addition, multiplication, subtraction, and magnitude comparisons. While originally applied to individual DM, Spiliopoulos et al. (2015) calculate the EIPs of popular decision strategies for normal-form games, and find that under time pressure players shift to strategies that are less complex, i.e., are comprised of fewer EIPs. Another class of heuristics that have been applied to strategic DM are decision trees, which structure the decision processes as a series of sequential operations conditional on the history of prior operations, eventually leading to a terminal node that determines the final decision. Empirical investigations of decision trees in the ultimatum game can be found in Fischbacher et al. (2013) and Hertwig et al. (2013).

The Adaptive-Toolbox paradigm (Gigerenzer and Selten 2002) posits that decision makers choose from a set of heuristics, and that a heuristic’s performance depends on the exploitable characteristics of the current environment. A decision maker is therefore faced with the task of how to match the appropriate heuristic to environmental characteristics. Obviously, any such choice will be affected by RT. How, in particular, are heuristics or strategies chosen if the decision maker has no prior knowledge of the relationship between heuristics’ performance and environmental characteristics? For individual DM tasks, Rieskamp and Otto (2006) find evidence that subjects use a reinforcement-learning scheme over the available heuristics. For strategic DM, Stahl (1996) concludes that subjects often apply rule-learning, which is essentially a form of reinforcement learning over a set of decision strategies. Closely related to this approach is the literature on evolution as the selection mechanism of decision rules, e.g., Engle-Warnick and Slonim (2004); Friedman (1991).

### Models of iterated strategic reasoning 

Models of bounded rationality incorporating finite iterated best responses, such as the iterated deletion of dominated strategies, cognitive hierarchy (Camerer et al. 2004) and Level-*k* models (Costa-Gomes et al. 2001; Stahl and Wilson 1995), make implicit predictions about RT. Although evidence in favor of these models has been based on choice data, there are falsifiable RT predictions that would provide further useful information. Cognitive hierarchy or Level-*k* models implicitly produce an ordinal ranking of RT over the degree of sophistication within a decision.^8^ For example, since Level-*k* agents must solve for the actions of all prior $k-1$ level players to calculate a best response, RT is a monotone increasing function of the level, *k*.

### Substantive models augmented with auxiliary assumptions

The joint modeling of choice and RT is not necessarily restricted to explicitly designed procedural models, but can be accomplished by redefining models of substantive rationality. For example, the EU maximization problem can be modified in the following ways:

#### The addition of constraints that capture cognitive costs, bottlenecks, and limitations to the standard maximization problem

The addition of a constraint to an unconstrained optimization problem can have RT implications if the constraint can be explicitly linked to time. For example, Matejka and McKay (2015) connect the Rational-Inattention model (Sims 2003, 2005, 2006) to the multinomial-logit choice rule often used to map the expected utility of actions to a probability distribution over the action space. The precision, or error parameter, in the multinomial-logit model is linked to the cost of information acquisition. Agents optimally choose the level of information they will acquire before making a decision.

#### Modification of the objective function

An alternative approach incorporating RT is based on the premise that the appropriate objective function in the wild is to maximize expected utility *per time unit*. This assumption is often used in evolutionary biology, where survival depends on the energy expenditure and intake per time unit, e.g., Charnov (1976).

#### The addition of auxiliary assumptions related to RT

Similarly to the discussion in Sect. 3.2, it may be possible to add auxiliary RT assumptions to substantive models (rather than heuristics) based on the information and operations required by the model, e.g., the number of parameters in a decision maker’s utility function. Recall from earlier discussions that in the context of social preferences this implies that a decision maker who is self-interested would exhibit lower RT than one who cares about an opponent’s outcome, since the latter requires the additional lookup and processing of their opponent’s payoffs.

### Search and attentional models

Models in this class explicitly account for information search or acquisition either externally (directly from the environment) or internally (from memory). For example, the Directed-Cognition model of external search (Gabaix and Laibson 2005; Gabaix et al. 2006) extends the agent’s objective function to include the opportunity cost of time, and is consistent with empirical evidence that subjects were partially myopic in their assessments of the future benefits and costs of additional information acquisition, thereby circumventing the intractability of a rational solution. Similarly, Bordalo et al. (2012) find that information salience is predictive of RT through the effect of salience on the allocation of attention. Internal information acquisition from memory is also time-dependent, e.g., memories that are more likely to be needed (are more recent and/or have been rehearsed more times) are retrieved more quickly (Schooler and Anderson 1997). In individual DM, Marewski and Melhorn (2011) leverage the explicit modeling of memory using the ACT-R framework (Anderson 2007; Anderson and Lebiere 1998) to infer which models are appropriate. In strategic DM, forgetting is found to constrain the strategies used by players in repeated games (Stevens et al. 2011).

### Sequential-sampling models

One of the main advantages of such models is the clear identification of the underlying process mechanism and the simultaneous modeling of both choices and RT. The instantaneous valuations of each available option are conceptualized as a deterministic drift component, which is a function of the expected payoff of the option, and a random component. Evidence for each option is accumulated over time, as determined by the drift rate and noise. The whole process resembles a random walk with a drift specified by the instantaneous valuations of each option. If there are no time constraints, then a decision is made when the accumulated evidence for any of the options reaches a threshold value. Intuitively, for a given threshold, a lower (higher) drift rate leads to a longer (shorter) mean RT. For a given drift rate, a higher threshold reduces the probability of erroneously choosing the option with the lower mean valuation as the effects of noise are diminished. Alternatively, if a time constraint is enforced, then rather than racing towards a threshold value, a decision is made in favor of the option that has the highest accumulated evidence at the time the constraint is reached.

Early work originated in the context of memory retrieval (Ratcliff 1978). Busemeyer and Townsend (1993) formalized this process for individual DM under risk (referred to as Decision Field Theory). Many variations and related models can be found in the psychology literature and more recently in economics (e.g., Busemeyer 2002; Caplin and Martin 2016; Clithero 2016; Fudenberg et al. 2015; Krajbich et al. 2010, 2012; Krajbich and Rangel 2011; Ratcliff and Smith 2004; Rieskamp et al. 2006; Smith 2000; Smith and Ratcliff 2004; Usher and McClelland 2001; Webb 2016; Woodford 2014). Although strategic DM can also be modeled in this manner, more complex characterizations of the decision processes are necessary. Spiliopoulos (2013) examines belief formation and choice in repeated games, extending a sequential model to capture strategic processes by assuming that the instantaneous drift is driven by an expected value calculation based on payoffs and strategic beliefs—the latter are determined by the retrieval of historical patterns of play from memory.

The first sequential-sampling models proposed a unitary-system model of behavior that can produce a variety of different behaviors by conditioning the decision threshold on the task properties and environment. Consequently, they were viewed as competitors to the dual-system approach, see Newell and Lee (2011). However, interesting hybridizations of dual-systems models and sequential-sampling models have been presented recently. Caplin and Martin (2016) propose a dual-process sequential-sampling model that first performs a cost-benefit analysis of whether accruing further information (beyond any priors) is expected to be beneficial, and then either makes an automatic decision based on the priors if the expected costs exceed the benefits or otherwise triggers a standard accumulation process. The discussion about the appropriateness of dual-system, sequential-sampling and hybrid models is ongoing and in our view deserves the attention it receives. The varying RT predictions of these competing models can be useful in model comparison and selection.

### Summary

We have presented a multitude of different models, often arising from opposing schools of thought, e.g., simple heuristics versus optimization under constraints, single versus dual-system models. The presented models also differ significantly in terms of whether they explicitly incorporate decision processes or address only the functional, e.g., according to Marr (1982), the former operates on the algorithmic and the latter on the computational (or functional) level. We are partial to models operating at the algorithmic level or what we refer to as procedural modeling—further discussed in Sect. 6.1. However, operating at a higher level of abstraction can also have benefits, including simplicity. We suspect that the type of model chosen for RT analysis will be highly dependent on a researcher’s proclivity; however, we encourage model comparisons between these different types of models. Furthermore, it may be the case that different types of models operate at varying degrees of time constraints; in this case we argue for a better understanding of the scope of these models and under what conditions each one is triggered in human reasoning.

## Benefits

### Improved external validity

In the Introduction, we expressed concerns regarding the external validity of standard experiments that do not account for time constraints and the opportunity cost of time by assuming virtually unlimited, costless information search and integration. We argue that external validity can be improved by increasing experimental control through RT experiments (discussed in Sect. 4.3), and that such experiments allow us to thoroughly investigate the speed–performance relationship (discussed in the following section), which is particularly relevant for decisions in the wild.

### Mapping the relationship between RT and performance

An often investigated relationship is the speed–performance or speed–accuracy trade-off. The difference between accuracy and performance is subtle but important. The former is a measure in the *choice* space, whereas the latter in the *consequence* space, which is essentially measured by the payoffs derived from a choice. For example, measures of accuracy include the proportion of actions that were dominated, the proportion that were errors (when clearly defined)—note that these measures do not capture the cost to the decision maker of said errors. However, if time is scarce or costly, *fast* errors may be optimal if they have a relatively small consequence on payoffs, and permit the allocation of time—and therefore reduced probability of an error—to decisions with higher payoffs.

A key insight of the ecological-rationality program (Gigerenzer 1988; Gigerenzer et al. 1999, 2011; Gigerenzer and Selten 2002; Ortmann and Spiliopoulos 2017) is that, in contrast to claims by researchers in the original adaptive decision maker tradition (Payne et al. 1988, 1993), more speed is not necessarily bought at the cost of lower performance. We note that this surprising result is conditional on an important methodological innovation, cross-validation, that only recently has found the appropriate appreciation in economics (e.g., Erev et al. 2017; Ericson et al. 2015)—see also Ortmann and Spiliopoulos (2017) for other references and details.

Economists seem well advised to thoroughly map the speed–performance relationship across classes of strategic games, and to do so possibly–at least for certain research questions–also by way of cross-validation. Obviously, for strategic DM it is necessary to define both the class of game and the strategies that opponents are using. Determining which classes of games we can expect realized payoffs to be negatively or positively related to time pressure or RT is an open question that seems worth investigating.

There exists less work on the speed–performance relationship compared to the speed–accuracy relationship in strategic DM, as researchers have focused on variables in the action space such as cooperation rates, error rates, degree of sophistication, or equilibrium selection. For example, Rubinstein (2013) finds a negative relationship between RT and (clearly defined) errors, but no relationship between RT and the consistency of behavior with EUT in individual DM tasks. However, an explicit discussion of whether RT is related to the actual performance of players is notably absent, albeit easily remedied. As discussed in “Appendix 2.2”, although a positive relationship between RT and the level of sophistication in reasoning seems intuitive and supported by the available evidence, in some games decreasing sophistication may actually lead to higher payoffs for all players of a game—recall the game in Table 5. Similarly, the findings by Guida and Devetag (2013) suggest that focal points are increasingly chosen under time pressure—in games where these focal points may help players to coordinate, this may result in higher payoffs but not necessarily so. We are aware only of three economics studies, already mentioned earlier, Kocher and Sutter (2006) in individual DM and Arad and Rubinstein (2012); Spiliopoulos et al. (2015) in strategic DM that explicitly relate performance to RT—more attention to the consequence space rather than the action space seems desirable.

If decision makers explicitly consider both performance and the necessary RT to achieve various levels of it, then an important unanswered question is how they choose the exact trade-off point (assuming a negative relationship exists between performance and RT)? Do they strategically choose this point conditional on task characteristics such as task difficulty, other concurrent cognitive load, types of time constraint, etc.? We present an indicative selection of hypotheses under the assumption that speed and performance are negatively related:

- Unconstrained Expected Utility maximization: The effect of RT is completely ignored, and subjects simply aim to maximize their expected utility.
- Unconstrained Expected Rate of Utility maximization: The objective function that is maximized is the expected utility per time unit.
- Performance satisficing: An aspiration level of performance (utility) is set, and RT is adjusted to keep performance constant.
- Time-constraint satisficing: A time-pressure constraint is externally set and is exhausted, thereby determining the performance.

We present some evidence from individual DM tasks for consideration. If the decision maker has the opportunity to repeatedly engage in the same task, then there exists a closed-form solution for the decision threshold that optimizes the *reward rate* for choice sets with two options (Bogacz et al. 2006). Hawkins et al. (2012) present evidence that subjects engage in performance satisficing rather than maximization. Satisficing requires the specification of how high the performance criterion is set, and how this may depend on prior experiences. Balci et al. (2010) find that subjects facing a two-alternative forced-choice task exhibit a bias towards maximizing decision accuracy rather than the reward rate initially, i.e., adopted a suboptimal speed–accuracy trade-off. However, after repeated exposure to the task subjects’ behavior moved significantly towards the maximization of the reward rate. Young subjects are more likely to seek a balance between accuracy and speed than older subjects; the former tend to maximize *reward rates*, especially with experience and extensive feedback, whereas the latter maximize* accuracy*, i.e., minimize errors (Starns and Ratcliff 2010).

### Explicit experimental control of RT

At first sight, experimental studies without any explicit exogenous constraint on RT may be immune from RT considerations. However, implicit time constraints may be inadvertently imposed by the experimenter or inferred by subjects. In consequence, studies that are otherwise similar may not be directly comparable if the implicit RT pressure varies across them. We conjecture that differences in implicit time pressure may drive some of the seemingly contradictory or non-replicable results in the literature if behavior is adaptive. Implicit time constraints may exist in many studies where RT is supposedly endogenous for the following reasons:

- Recruitment materials usually mention the expected length of the experiment, which is likely to cue subjects to the experimenter’s expectation of the time it takes to complete the task.
- Experimental instructions often include information that may influence the amount of time a subject decides to allocate to tasks. Strategic interaction of subjects, for example, might imply a weak-link matching scheme where the slowest player determines the time the session takes.
- For practical reasons–such as random matching for the determination of payoffs, or to avoid disturbances from subjects exiting early–subjects might have to wait for all participants to finish before they are allowed to collect payment and leave. Similarly, subjects may be delayed whilst waiting for other subjects to enter their choices before moving on to the next round of a repeated game.
- Subjects may be affected by many subtle cues in the wording of instructions. Benson (1993) and Maule et al. (2000) are cautionary tales of the effects of instructions on *perceived* time pressure—behavior was significantly influenced by different (loaded) instructions describing the same objective time limit.

Concluding, the loss of experimental control associated with implicit time constraints is a potential problem. Consequently, experiments with explicit exogenous time constraints may be significantly more comparable–and less noisy within a particular experiment–as they do not run the risk of participants subjectively inferring implicit time pressure. Alternatively, the adverse impact of implicit time constraints can be reduced without imposing explicit time constraints by permitting subjects to engage in an enjoyable activity, e.g., surf the internet if they have completed all their tasks early.^9^ We would also encourage accounting for implicit time constraints in meta-analyses of studies–to the best of our knowledge this has not been done before.

### Improved model selection, identification and parameter estimation

Model selection and identification, as we have argued earlier, can be sharpened by the use of RT. Models differ in their explanation of how an adaptive decision maker will react to time constraints and, ultimately, how observed behavior will change. As mentioned, differential RT predictions are a valuable aid in comparing competing models of behavior, e.g., Bergert and Nosofsky 2007; Marewski and Melhorn 2011. Significant information can be gleaned from the relationship between RT and candidate variables of observed behavior, such as the error rate, realized choices, adherence to theoretical concepts such transitivity, equilibrium concepts etc. In short, models that make RT predictions in addition to choice are more structured, rendering them more falsifiable as both RT and/or choice data can refute them.

### Classification of heterogeneous types

RT data can sharpen the classification of subject types, particularly in cases where two or more different decision strategies prescribe the same, or very similar, observed choices. The Allais-Paradox task in Rubinstein (2013) is a case in point—patterns of choices differed significantly between subjects with low and high RT. Another example involves distinguishing between two types of learning: (a) incremental learning, where RT is expected to be smoothly decreasing with experience, and (b) eureka or epiphany learning, where RT should abruptly fall when subjects have an important insight that has a lasting impact on play (Dufwenberg et al. 2010; McKinney and Huyck 2013; Schotter and Trevino 2014b).

### RT as a proxy for other variables

RT may be used as a proxy for effort (e.g., Ofek et al. 2007; Wilcox 1993) to examine the effects of variations in important variables such as experimental financial incentives, labor productivity incentives, and other general incentive structures. For example, RT can be used as a proxy for effort in the debate regarding financial incentives in experiments. A positive relationship between RT and the magnitude of financial incentives, ceteris paribus, would support the viewpoint that incentives matter. Alternatively, RT may also be a proxy for the strength of preference for an option—see the empirical evidence (e.g., Chabris et al. 2008, 2009) in favor of a negative relationship between RT and the difference in the strength of preference among available options. Such a relationship is also predicted by the sequential-sampling models discussed in Sect. 3.6.

## Challenges

### Identification 

The use of RT–above and beyond choices only–is beneficial for identification purposes, however it is not a panacea. Recall the extensive discussion in Sect. 2.1.2 about reverse-inference and identification in games where social preferences are important. The interaction of players in strategic DM provides an additional layer of complexity in the identification of processes, e.g., beliefs may play an important role. Consider social-dilemma games where RT constraints are implemented to examine their causal effect on the degree of cooperation or pro-social choices. If it is common knowledge that all players face time pressure in a treatment, then players may change their beliefs about how cooperative their opponents will be. Consequently, changes in social preferences and beliefs would be confounded, rendering the attribution to either impossible. These issues can be alleviated by careful choice of experimental design and implementation details, and the concurrent collection of other process measures such as information search and beliefs. For example, Merkel and Lohse (2016) explicitly collect players’ beliefs about their opponents’ likely behavior across different time treatments.

Identification may also be hampered in cases where RT constraints have a differential effect on other treatments, i.e., when RT interacts with the other treatments. For example, consider a public good experiment played under time pressure, where the treatments manipulate the number of players (few versus many). If increasing the number of players makes the game more complex or difficult, then a specific level of time pressure may have a greater relative impact in the treatment with more players. Such cases are easily remedied with an appropriate full factorial $2\times 2$ design where RT (endogenous versus time pressure) and the number of players (few versus many) are both manipulated, as the main effects of each factor and their interaction can then be recovered.

### Irregular RT distributions, outliers and non-responses

The question of whether extreme values are regarded as outliers or not, and if so, how they are handled in the data analysis is of considerable importance and consequence—recall the debate in Rand et al. (2012), Tinghög et al. (2013) reviewed earlier. Very short RTs may arise from fast-guessing, or very long RTs from subjects that are not exerting much effort and are bored.^10^ Furthermore, the use of time pressure often leads to a number of non-responses if subjects do not answer on time. This leads to a selection problem if non-responses are correlated with subject characteristics. How these RT idiosyncrasies are treated is of paramount importance.

Consequently, endogenous RT distributions tend to be non-normal (left-truncated at zero), heavily skewed and often consist of extreme (low and high) values. This renders analyses using mean RT and ANOVA problematic. Whelan (2010) recommends the use of the median and inter-quartile ranges for such cases, but notes that since true population medians are strongly underestimated for small sample sizes, median RTs should not be used to compare conditions with different numbers of observations. Another common solution is to appropriately transform the RT distribution into an approximate normal distribution, usually through the use of a log-transform. Outliers can have a significant impact on parametric summary statistics; possible solutions include using (a) robust non-parametric statistics, (b) Student *t*-distributions that allow for fat-tailed distributions (e.g., Spiliopoulos 2016), and (c) hierarchical modeling (see Sect. 6.3). We refer the reader to Van Zandt (2002) and Whelan (2010) for an extensive discussion of RT distribution modeling.

### Heterogeneity

Experimental studies of individual DM and strategic DM generally find significant between-subject heterogeneity, e.g. in learning models (Cheung and Friedman 1997; Daniel et al. 1998; Ho et al. 2007; Rapoport et al. 1998; Rutström and Wilcox 2009; Stahl 1996; Shachat and Swarthout 2004; Spiliopoulos 2012).

Between-subject heterogeneity can be attributed to two sources. *Parameter heterogeneity* arises from subjects using the same type of model, i.e. identical functional forms, but with individual-specific parameters. *Model heterogeneity* arises from subjects using completely different models, e.g. heuristics that cannot be nested within a more general model.

It is imperative to model heterogeneity directly as pooling estimation affects parameter recovery and model selection (Cabrales and Garcia-Fontes 2000; Cohen et al. 2008; Erev and Haruvy 2005; Estes and Maddox 2005; Estes 1956; Wilcox 2006). Consequently, econometric models of RT should also allow for different RTs across subjects and heterogeneous effects of various RT determinants (e.g., Spiliopoulos 2016). Modeling both parameter and model heterogeneity requires the estimation of both finite mixtures and random-effects or hierarchical econometric models presented in Sect. 6.3. See our working paper (Spiliopoulos and Ortmann 2016) for an extended discussion.

### RT measurement error

Experimentalists will typically delegate RT data collection automatically to whatever software package they use to set up the experiment, or in rarer cases may code their own experiment from scratch. While the accuracy of RT data collection has not been extensively examined in economics, more work has been done in psychology. We note that in psychology response times are often on the order of hundreds of milliseconds compared to seconds in economics. Therefore the accuracy of data collection for such finer graduations is not as important in experimental economics. Variations in RT estimates can be caused by any combination of hardware, software, and network latencies (for online experiments). The importance of these variations depends on their magnitude relative to the absolute RTs in the experiment and whether they are systematic or random, i.e., whether the noise can be expected to average out for a large enough number of observations. The general conclusion is that while absolute measures of RT may not be reliable across differences in these three sources of noise, relative measures of RT remain relatively faithful. Furthermore, the standard deviation of the induced noise is very low compared to the scale of RTs that experimental economics deals with.^11^

The most popular experimental economics software is z-Tree (Fischbacher 2007) and it includes the ability to internally measure response times. Perakakis et al. (2013) propose an alternative method based on photo-sensors that capture changes in the presentation of information on screen to counter-act the possible mis-calculations arising from the computer’s internal timing. Their photo-sensor system recorded response times that were on average 0.5 s lower than those recorded internally by z-Tree. While this difference may be problematic if the study attempts to link the timing of events with other biophysical markers such as heart-rate, it did not adversely affect the conclusions drawn from RT analyses across treatments. In economics, we will typically be interested in relative RTs and changes across treatments rather than absolute RTs; therefore, z-Tree should be accurate enough for the vast majority of applications. Seithe et al. (2015) introduced a new software package (BoXS) specifically designed to capture process measures in strategic interactions including RT. They present evidence that this software’s RT accuracy is approximately $\pm 50$ ms (when presenting information for at least 100 ms), which is more than adequate for economic applications.

Concluding, for online experiments we can expect significant variations arising from both hardware and network sources, i.e., RT measurements will be relatively noisy. However, online experiments usually have a large number of subjects so that the noise often cancels out. For experiments in the laboratory, there does not seem to be a significant problem in the accuracy of RT measurements in the most common types of applications.

## Desiderata

### Procedural modeling

The existing RT literature is dominated by non-procedural (descriptive) rather than procedural modeling. We believe that in many instances procedural models are more useful than non-procedural models, as the former allow for comparative statics or quantitative predictions regarding the *joint *distribution of choice and RT. Such models can be falsified either by incorrect choice or RT predictions, thereby increasing the statistical power of experiments and associated hypothesis tests. Other process measure variables discussed in the next section could increase the power even further. Procedural models also provide a coherent framework within which to organize and define exactly *how* behavior adapts to time constraints—various mechanisms are discussed in “Appendix 2”.

### Concurrent collection of other process measures

Few existing studies in experimental economics collect other process measures beyond choice and RT, some notable exceptions can be found in Table 3. Examples of other decision or process variables include information search using Mouselab or eye-tracking techniques (Crawford 2008), response dynamics (Koop and Johnson 2011), provisional choice dynamics (Agranov et al. 2015), belief elicitation (Schotter and Trevino 2014a), communication between players (Burchardi and Penczynski 2014), verbal reports (Ericsson and Simon 1980), physiological responses and neuroeconomic evidence (Camerer et al. 2005). However, it should be kept in mind that collecting these process measures is more disruptive than RT, and therefore their collection could influence behavior. The reader is referred to Glöckner (2009) and Glöckner and Bröder (2011) for examples of procedural models predicting multiple measures: RT, information search, confidence judgments, and fixation duration. Other examples of the value added of process measures beyond choice data include Johnson et al. (2002), Costa-Gomes et al. (2001) and Spiliopoulos et al. (2015).

### Hierarchical latent-class modeling

Hierarchical latent-class models can be an effective ally in capturing heterogeneity and outliers in the data. Estimating models per individual–whilst capturing individual heterogeneity–may not be the best line of attack due to the large number of free parameters and susceptibility to overfitting. Instead, we propose hierarchical latent-class models that capture both types of between-subjects heterogeneity with a reduction in free parameters (Conte and Hey 2013; Lee and Webb 2005; Scheibehenne et al. 2013; Spiliopoulos 2012; Spiliopoulos and Hertwig 2015). The latent classes capture model heterogeneity, whereas the hierarchical structure models parameter heterogeneity.^12^ The latent-class approach yields both prior and posterior (after updating the prior with the observed data) probabilities of subjects belonging to the specified latent classes. An additional bonus to such an econometric specification is that outliers can automatically be identified as belonging to one of the classes. Furthermore, latent-class models can also be used for within-subject heterogeneity (Davis-Stober and Brown 2011; Shachat et al. 2015), e.g., the adaptive use of heuristics.

### Cross-validation

Models of choice and RT ought to be subjected to the same strict demands that we impose on existing models. Specifically, procedural models should be competitively tested on out-of-sample data to ascertain their predictive ability or generalizability (Ahn et al. 2008; Busemeyer and Wang 2000; Yechiam and Busemeyer 2008), such as in the context of a large-scale model tournament (Ert et al. 2011; Spiliopoulos and Ortmann 2014). A commonly used technique is that of cross-validation, which requires that experimental data be partitioned into an estimation and a prediction dataset. Models are then fitted on the estimation dataset, and their performance is judged on the prediction dataset. This technique is effective in comparing models of varying flexibility as it avoids the problem of over-fitting by complex models. This is, in fact, the crux of one of the main tenets of the ecological-rationality program (Gigerenzer 1988; Gigerenzer et al. 1999, 2011; Gigerenzer and Selten 2002)—for a critical review see Ortmann and Spiliopoulos (2017). Simple heuristics can outperform complex decision models on unseen data exactly because they have less ability to overfit to noise or uncertainty in the environment.

Interestingly, cross-validation has not been extensively used in the RT literature with the exception of a few notable studies. Chabris et al. (2009) and Schotter and Trevino (2014b) find that RT can be predictive of intertemporal choice and behavior in global games, respectively. Importantly, Rubinstein (2013) and Rubinstein (2016) find evidence that RT can be predictive of both individual DM and strategic DM choices across tasks. These results suggest that the degree of mutual information in choice and RT data is significant and can be exploited—we hope to see more analyses of this kind.

### Experimental implementation

We have argued that we expect significant between-subject heterogeneity in strategic DM. In conjunction with the inherent stochasticity of choice and the practical limitations on the number of observations from empirical studies, this will make inference challenging. In some cases, an effective solution in terms of experimental implementation is to design within-subject treatments, thereby eliminating the between-subjects source of variability. However, the decision to use a between- or within-subject design implies a trade-off (Charness et al. 2012); a within-subject design will limit the number of tasks that can be examined.

The RT literature using individual DM and strategic DM tasks has so far used rudimentary designs where behavior is observed as a function of different RT constraints, thereby revealing the RT-behavior profile. Existing experiments often use a *t*-treatments design, where *t* is the number of treatments with different time constraints and is usually equal to two or three. We will argue that we can further augment the usefulness of RT data by expanding experimental designs by, firstly, increasing the number and type of time treatments and, secondly, by varying another variable, namely the difficulty of the task. What we propose is a $t\times d$ factorial design ($d=$number of treatments with varying difficulty) where the manipulation of the time constraint reveals the speed–performance profile and manipulation of task difficulty reveals shifts of this profile. Task difficulty can be defined in various ways, which may differentially affect the speed–performance profile. Some examples of defining characteristics of difficulty for strategic DM are:

1. The size of players’ action sets
2. The number of players in a game
3. The distance between a player’s (subjective) expected payoffs per action
4. The uncertainty about opponents’ types
5. The existence of imperfect information
6. The lack of focal points
7. The presence of cognitive load

Moving beyond the two-dimensional speed–performance trade-off to a three-dimensional speed–performance-difficulty profile, will generate more conflicting predictions between models, thereby aiding model comparison and identification. Also, note that an experimental design that explicitly manipulates difficulty addresses one of the critiques put forth by Krajbich et al. (2015); namely, that differences in task difficulty and the degree that behavior is instinctive may be confounded in existing studies that do not control for the former. We are aware that this line of attack might run into hostile budgetary defense lines.

Finally, researchers should consider when and how to disclose a time-constraint to subjects. If RT is exogenous, then specifics of the constraint can be announced in advance, or revealed to subjects during the decision process. For example, subjects may not be told how long they will have to make a decision but may be alerted in real-time when they must decide. Knowledge of the constraint may induce a subject to significantly change their decision process, e.g., under extreme time pressure they may switch to a simple heuristic, or in the dual-system approach, they may switch from the deliberative and slow System 2 to automatic and fast System 1 (Kahneman 2011). If subjects do not know what the time limit will be, they may respond in the following two ways: (a) be more likely to use the same decision process and simply terminate when the time limit is announced and (b) make a provisional fast decision and then search for a better alternative, as in Agranov et al. (2015).

## Discussion

We have presented the state of the art of response time (RT) analysis in cognitive psychology and experimental economics, with an emphasis on strategic decision making. Experimental economists have only recently directed attention to RT, in stark contrast to experimental psychologists. A comparison of the methodology of these two groups exposes an important difference—experimental psychologists predominantly use procedural models that make explicit predictions about the joint distribution of choice and RT, while experimental economists predominantly restrict their analyses to descriptive models of RT. We offered arguments regarding the advantages of RT analysis, particularly in conjunction with procedural modeling. We are specifically concerned that investigating decision making in the lab without any time-pressure controls, or at least an explicit opportunity cost of time, might limit the external and ecological validity of experiments. In our view, this void in the literature deserves more attention than it has attracted. We envision significant advances in our understanding of behavior and its relationship to RT. Our assessment of the potential of RT analysis is partially inspired by results in cognitive psychology and we recommend a research agenda for strategic DM that parallels that of individual DM.

At the very least, there is no reason *not* to collect RT data for computerized experiments as their collection can be done without disrupting or influencing the decision process and is costless. RT data provides further information improving model selection, the identification of decision processes and type classification. The collection of RT data is, of course, not a panacea: although it increases our ability to identify models and decision processes, it does not necessarily provide full identification. Furthermore, there are some important challenges that experimental economists face; many of these challenges are unique to strategic decision making, arising from the complex interaction of agents. We presented desiderata aimed at addressing these challenges and unlocking the potential of RT analysis.

We have discussed the multitude of ways that strategic players can adapt to time constraints (and offer a formal framework in “Appendix 2”). For example, players may change how they search for information, how they integrate information, and how they adapt their beliefs about the strategic sophistication of opponents. Our discussion of the models in the literature concludes that most models need to be extended in new ways to fully capture these possibilities.

The majority of the literature has been devoted to investigating the relationship between social preferences and response time. There is an opportunity for new important work in non-social dilemma games, especially repeated games. Less researched, yet in our eyes, important topics for future work on RT include a thorough investigation of the speed–performance profile, and the relationship between emotions and RT. For the former, important questions include how people decide to trade off speed and performance, and how they allocate time to a set of tasks. For the latter, the role of emotions, such as anger, regret and disappointment, and their effects on response time seem worth further study.

Concluding, we anticipate (and already see realized some of the predictions we made in our 2013 version of this paper) that explicit modeling of RT data will provide important insights to the literature, especially in conjunction with other non-choice data arising from techniques that turn latent variables of processes into observable variables, e.g., belief elicitation and information search. Extending experimental practices to include RT and time–pressure controls is an important step in the study of procedural rationality and adaptive behavior in an externally and ecologically valid manner.

## Appendix 1: Literature review

Table 3 presents RT studies on strategic DM sorted into two categories, first published studies and then working papers. Within each category we ordered studies chronologically and within each year alphabetically. We also classify studies along the following dimensions: (a) whether RT was endogenous (*en*, i.e., no time constraints) or exogenous (*tp* = time pressure, *td* = time delay), (b) the type of model used to explain behavior, (c) the type of task, (d) whether other variables were measured, and (e) the main conclusions.

**Table 3:** RT literature ordered by publication status (published versus unpublished), followed by chronological and alphabetical order (RT classification:*en* endogenous, *tp* time pressure, *td* time delay)

Studies	RT	Model	Conclusions	Other measures	Task
**Published works**					
Brañas-Garza et al. (2016)	*en*	Moral, iterated/strategic reasoning	RT was longer in the Ultimatum game compared to the Yes-no game (due to greater strategic risk and the lack of a dominant strategy), subjects with longer RT exhibit more dispersed behavior (due to the resolution of moral dilemmas)		Ultimatum, Yes-no games
Cappelen et al. (2016)	*en*	Dual-system	Fair behavior is associated with shorter RT even after controlling for cognitive ability and other controls	Progressive matrices test	Dictator game
Devetag et al. (2016)	*en*	Dual-system, heuristics, search and attentional	The number of lookups (and lookup time) for different cells is predictive of choice and strategic thinking	Eye-tracking	$3\times 3$ normal form games
Nishi et al. (2016)	*en*	Cognitive dissonance/conflict	Reciprocal choices are faster than non-reciprocal choices. Cooperation is faster than defection in cooperative environments and vice-versa in non-cooperative environments		Repeated public goods and Prisoner’s dilemma games
Rubinstein (2016)	*en*	Dual-system	Actions defined as contemplative or instinctive using RT can be used to create a player typology based on how contemplative a player is. This typology is found to be predictive of behavior in out-of-sample games		Ten different games
Agranov et al. (2015)	*en*	Iterated/strategic reasoning	Large proportion of naive types, whose players often switch choices haphazardly over time, without exhibiting increasing sophistication. Strategic players switch choices less and their behavior increases in sophistication over time		2/3 guessing game (p-beauty)
Evans et al. (2015)	*en,tp,td*	Dual-system, cognitive dissonance, sequential sampling	Greater decision conflict is associated with slower RT, leading to an inverted-U shaped relationship between RT and cooperation	Questionnaire	Prisoner’s dilemma, one-shot and repeated public goods games, trust game
Oechssler et al. (2015)	*td*	Emotions	Time delay (24 hours) leads to a lower rejection rate for lottery payoffs, but no difference for cash payoffs	Cognitive Reflection Test	Ultimatum game
Rand et al. (2015)	*tp,td*	Dual-system	Political group membership does not interact with time constraints in affecting cooperation levels		One-shot public goods game, continuous Prisoner’s dilemma
Cone and Rand (2014)	*tp,td*	Dual-system	Cooperation is higher under time pressure when the PG game is framed in a competitive versus cooperative context; thereby excluding adherence to social norms as an explanation		One-shot public goods game
Eliaz and Rubinstein (2014)	*en*	Emotions	No differences in RT for different types of subjects		Fairness of randomization procedures in social situations
Lindner (2014)	*tp*	Iterated/strategic reasoning	Entry rates under time pressure are lower the more steps of reasoning used by subjects		Market entry game, 11–20 Money Request Game
Rand et al. (2014)	*tp,td*	Dual-system	Time pressure induced more cooperation on average; however, cooperation under time pressure decreased with experience		One-shot public goods game
Rand and Kraft-Todd (2014)	*tp,td*	Dual-system	Time pressure increased cooperation rates only for subjects that were trusting and inexperienced. Changes in the cooperation rate under time pressure were similar for both competitive and collaborative framing of the game	Questionnaire (degree of trust and faith in intuition)	One-shot public goods game
Verkoeijen and Bouwmeester (2014)	*tp,td*	Dual-system	No significant effect of time pressure, knowledge of opponents’ moves, type of opponent (human versus computer) on contribution levels		One-shot public goods game
Guida and Devetag (2013)	*en*	Dual-system, heuristics	RT is less for games with a focal point, is positively correlated to the variance of the action with the highest average payoff, is higher the more equilibrium actions taken by a subject	Personality test, Holt-Laury lottery task	$2\times 2$ and $3\times 3$ normal form games
Fischbacher et al. (2013)	*en*	Social preferences /heuristics	RT is increasing in the number of social properties of the allocations that heterogeneous subjects attended to, e.g., kindness, fairness		Mini-ultimatum games
Hortala-Vallve et al. (2013)	*tp*	Cognitive effort	Better plans of action were pursued the longer RT was, the relationship between RT and the (in)completeness of information depends on the bargaining procedure		Bargaining
Jiang (2013)	*en*	Cognitive effort	Honest choices exhibit longer RT		Cheating games
Lindner and Sutter (2013)	*tp*	Iterated/strategic reasoning, cognitive effort	Subjects’ behavior was closer to the equilibrium prediction under time pressure		11–20 Money Request Game
Lotito et al. (2013)	*en*	Dual-system	RT and own contributions are negatively related, RT and others’ recent contributions (i.e., degree of prior cooperation) are negatively related		Public goods game
McKinney and Huyck (2013)	*en*	Eureka learning, heuristics	35% of subjects experienced a Eureka moment–i.e., realizing that a heuristic works well–after which RT is consistently less		Nim game
Neo et al. (2013)	*td*	Dual-system	No relationship between time delay (15 mins.) and sender/responder choices (IG), time delay associated with decreased rejections by responders (UG)		Investment (IG) and ultimatum (UG) games
Rubinstein (2007)	*en*	Dual-system	Actions categorized as instinctive were executed more quickly than actions categorizes as cognitive, i.e., requiring a deeper reasoning process		Numerous games and non-strategic tasks
Tinghög et al. (2013)	*tp,td*	Dual-system	No effect of time pressure on cooperation rates		One-shot and repeated public goods and prisoner’s dilemma games
Arad and Rubinstein (2012)	*en*	Iterated/strategic reasoning	Longer RT was associated with higher average payoffs		Colonel Blotto game
Glazer and Rubinstein (2012)	*en*	Iterated/strategic reasoning	The more difficult it is to be persuasive, the longer the RT		Persuasion game
Rand et al. (2012)	*tp,td*	Dual-system	The level of contributions is inversely related to RT (whether endogenous or exogenous)		One-shot public goods game
Cappelletti et al. (2011)	*tp*	Dual-system	Proposers under time pressure offer more, however there is no effect of cognitive load on offers		Ultimatum game
Grimm and Mengel (2011)	*td*	Dual-system, emotions	Time delay (10 mins.) increases the acceptance rate of low offers if subjects had not explicitly or implicitly expressed negative emotions		Ultimatum game
Matthey and Regner (2011)	*en*	Cognitive dissonance	RT is longer in social dilemmas where cognitive dissonance plays a role—in this case, when subjects can choose whether to observe the other player’s payoffs or not		Dictator game
Suter and Hertwig (2011)	*tp*	Dual-system, cognitive effort	Time pressure reduces cognitive control over moral instincts, thereby increasing the probability of deontological responses		Moral dilemmas
Gneezy et al. (2010)	*en*	Cognitive effort	RT is lower in winning positions than losing positions, also RT increases the more steps required in backward analysis of the game		Race game
Pintér and Veszteg (2010)	*en*	Dual-system	No relationship between RT and truthfulness		Voting schemes
Ibanez et al. (2009)	*tp*	Cognitive effort	Time pressure resulted in less search (sub-optimal) in the short-run (but no long-term effect), RT declined with experience without a performance trade-off		Bid/search task
Kuo et al. (2009)	*en*	Dual-system	RT was lower for coordination games compared to dominance-solvable games	fMRI	Dominance solvable (DS) and coordination (C) games
Piovesan and Wengström (2009)	*en*	Social preferences, emotions	Lower RT is associated with selfish choices both between- and within-subjects		Dictator game
Knoch and Fehr (2007), Knoch et al. (2006)	*en*	Self-control, fairness	Disruption of the right PFC using rTMS was associated with lower RT and a greater rate of acceptance of unfair offers	rTMS, elicitation of judgments	Ultimatum game
Rubinstein (2007)	*en*	Dual-system	Significant heterogeneity in RT was found conditional on the responses, lower RT was associated with more intuitive choices and less sophisticated reasoning		Normal form, p-beauty, ultimatum games
Kocher and Sutter (2006)	*tp,en,inc*	Iterated/strategic reasoning	High time pressure results in lower payoffs and slower convergence to the equilibrium, RT is lower for incentivized time-dependent payoffs without a negative impact on performance		p-beauty games with continuous payoffs
Sutter et al. (2003)	*tp*	Emotions	Responders are more likely to reject offers under time pressure; however, this difference disappears with repetition		Ultimatum game
**Unpublished works**					
Goeschl et al. (2016)	*en,tp*	Dual-system	Subjects contributed less under time pressure—this effect was stronger for subjects that were not confused about the game, i.e., where not error-prone. However, they did not find more confusion about the game in a time-pressure treatment		One-shot and repeated public goods game
Merkel and Lohse (2016)	*en,tp,td*	Dual-system, sequential sampling	Accounting for the subjective difficulty of choosing between alternatives, within- and between-subjects evidence did not confirm the hypothesis that time-pressure leads to fairer decisions		Dictator and Prisoner’s dilemma game
Spiliopoulos (2016)	*en*	Heuristics, dual-system	RT depends on the interaction between the win-stay/lose-shift heuristic and a pattern-detecting reinforcement learning model, conflict leads to longer RT		Repeated constant-sum game
Dyrkacz and Krawczyk (2015)	*tp*	Dual-system	Under time pressure, subjects were more averse to inequality (if their own payoff was less than their opponent’s)		Dictator and other games involving social preferences
Karagözoğlu and Kocher (2015)	*tp*	Heuristics, focal points, fairness	Under high time pressure, disagreement rates increase and last-minute agreements are more frequent; the probability of settling on an explicit reference point increases at the cost of the implicit equal-split		Bargaining
Recalde et al. (2015)	*en*	Errors	Lower RT is associated with erroneous decisions (dominated choices), faster subjects were insensitive to changes in payoffs whereas slower subjects were more sensitive		One-shot and repeated public goods game
Spiliopoulos et al. (2015)	*tp*	Iterated/strategic reasoning, heuristics	Under time pressure, subjects are more likely to not search for opponents’ payoffs, and there is a significant increase in the use of the Level-1 heuristic accompanied by a reduction in the Dominance-1 heuristic and Nash equilibrium behavior	Information search (Mouselab)	One-shot $3\times 3$ normal form games
Turocy and Cason (2015)	*en*	Incentives	RT tends to be longer for higher signals in first-price auctions, RT is independent of signals in second-price auctions		First- and second-price auctions
Schotter and Trevino (2014b)	*en*	Search and attentional, eureka learning	RT can be used to predict out-of-sample behavior with more accuracy than the equilibrium prediction		Global games
Halali et al. (2011)	*en*	Dual-system, cognitive control	RT is less when rejecting an unfair offer than accepting it, ego-depletion led to an increase in the rate of rejection of unfair offers		Ultimatum game
Bosman et al. (2001)	*td*	Emotions	Behavior and self-reported emotions are not affected by a time delay of 1 h	Elicitation of expectations and emotions	Ultimatum game

## Appendix 2: A framework for time-constrained strategic behavior

We present a framework for organizing the multitude of ways that decision makers can react to time constraints. Our framework allows us to first ask *which* decision processes are adjusted in response to time constraints, and subsequently *how* these decision processes are adjusted. To accomplish this we first categorize different types of decision processes by the function they perform. Subsequently, we define and relate specific ways of adjusting to these categories of processes. We are guided by existing results for individual DM tasks. Miller (1960) hypothesized that DMs react to time pressure–or more generally any type of information overload–in several ways.^13^ We present the four main time-pressure adaptations from Miller (1960) that we deem most important and that have been robustly verified in the individual DM literature. We extend these specifically to strategic DM tasks and suggest other ways of responding to time pressure that are unique to strategic DM. In our working paper (Spiliopoulos and Ortmann 2016, Table 8) we provide a more detailed comparison of RT analysis across three types of tasks, judgment, individual DM and strategic DM.

### Decision processes

A complete procedural model should describe how relevant information is acquired and processed to arrive at a decision. The dynamics of the information-search and -integration processes (including stopping rule) characterize the time required to reach a decision. Reaction time is often modeled as the sum of two main components: the decision component and the non-decision component. To model strategic DM it is useful to break down these two components into sub-processes and their associated response times.

The decision RT component consists of the following sub-processes:

#### Information-acquisition sub-processes

These processes require time to search for and acquire information. We further divide the acquisition of information into internal and external search. External search involves the real-time acquisition of information (stimuli) from the environment. Internal search is the retrieval of information stored in the memory system.

#### Strategic sub-processes

Significant time is also required to implement strategic processes (deliberative), such as analyzing a game and forming beliefs about an opponent’s behavior (or level of sophistication), elimination of dominated actions, et cetera.

#### Information-integration sub-processes

Time is required to compare and integrate the available information regarding choices. The input to these processes is the output of the information-acquisition sub-processes, and in the case of strategic DM may also include outputs from the strategic sub-processes if they transform other acquired information.

The non-decision RT component consists of:

#### Motor function response

Executing the required motor functions to indicate or implement a response also requires time.

Response time is therefore conceptualized as a function of the time required to complete the relevant sub-processes discussed above. The simplest function would be additive and separable; however, the assumption of separability, or independence, of these four components is tenuous. Non-separable processes may be an efficient use of limited cognitive resources and time.

### Adaptation to time constraints

We present some robust findings from the psychology literature on the adaptation of decision sub-processes to time pressure. These are primarily derived from individual DM tasks, consequently they are useful for thinking about how information-acquisition and -integration processes may be affected in strategic DM tasks, when strategic processes do not matter (much). Finally, we conjecture the existence of adaptations that are specific to strategic DM tasks, and have yet to be thoroughly investigated in the literature. Table 4 presents our framework in a tabular format, relating decision processes to the type of adaptation, and to the types of models that can account for each adaptation. Since we have not yet presented the types of models extensively, the reader might want to return to this table after reading Sect. 3.

**Table 4:** A framework for time-constrained adaptive behavior

	Strategic DM		
	Individual DM		
	Information acquisition	Information integration	Strategic processes
Acceleration	$\checkmark$	$\checkmark$	$\checkmark$
Filtration			
Fewer options	$\checkmark$ (SA, LEX)		
Fewer contingencies	$\checkmark$ (SA, LEX)		
Weighting of information		$\checkmark$ (SA)	
Focality enhancement		$\checkmark$ (DS)	
Memory effects	$\checkmark$ (SA)		
Strategy shift	$\checkmark$ (ADT,ISR)	$\checkmark$ (ADT,ISR)	$\checkmark$ (ADT,ISR)
Criteria shift		$\checkmark$ (SSM)	
Iteration reduction			$\checkmark$ (ISR)

All models are described in detail in Sect. 3: *SA* Search and attentional models, *LEX* lexicographic heuristics, *DS* dual-system, *ISR* iterated strategic reasoning models, *SSM* sequential-sampling models, *ADT* adaptive toolbox, *SMA* substantive models with auxiliary assumptions

#### Acceleration

The existing decision sub-processes are performed more quickly without changing strategy at the possible cost of introducing errors (Ben Zur and Breznitz 1981; Edland 1994; Kerstholt 1995; Maule et al. 2000; Payne et al. 1988).

#### Filtration

Priority and attention to information acquisition and processing is given selectively to information that is perceived as more important (Maule et al. 2000). At the information-acquisition stage, filtration can be manifested in games as the retrieval of information from *fewer options* (i.e., a player’s own actions in the game) or *fewer contingencies* (i.e., an opponent’s actions in the game); this may be observed as fewer lookups or less gaze time per piece of information. Under time pressure, the predominant effect for individual DM tasks is a shift from alternative- to attribute-based search and processing.^14^ At the information-integration stage, filtration affects the *weighting of information* in the integration process. Initial evidence on filtration found that negative information was relatively more important than positive information (Ben Zur and Breznitz 1981). However, the robustness of the direction has been contested and may be context-dependent. For example, Edland and Slovic (1990) find that filtration leads to greater weighting of positive information relative to negative information, whereas Maule and Mackie (1990) find no significant shift in relative importance. Note, any relative shift in attention between positive and negative information has important implications for the risk-taking behavior of individuals. Finally, we hypothesize another adaptation that we term *focality enhancement*. Under significant time pressure, subjects may attend more to information that has focal properties, e.g., larger payoffs, actions singled out by social norms, et cetera. *Memory effects* may also play an important role under constrained RT. Memory encoding and retrieval may be adversely affected, e.g., the number of items that are held in short-term memory may be restricted further beyond the proposed limit of $4\pm 1$ items (Cowan 2000), which is an update from the $7\pm 2$ items argued for in Miller (1956).

#### Strategy shift

Strategy shift is a change in the type of decision strategy selected. An adaptive decision maker could, for example, choose a heuristic that is effective for the current environment or one that is feasible given time constraints. In strategic DM a player under time pressure may change her strategy because of (a) a change in their belief about an opponent’s likely strategy if he is also under time pressure or (b) insufficient time to execute the preferred strategy. Consequently, time pressure may cause a disconnect between the *potential* sophistication of a player and the *realizable* sophistication. For example, assume two players are engaged in the following normal-form game (Table 5) and that both players are capable of using a Level-2 (L2) heuristic (Costa-Gomes et al. 2001; Stahl and Wilson 1995).^15^ Assume next that due to time pressure they are only able to implement the less demanding Level-1 heuristic. In Table 5, we denote the joint outcomes if players were to use a variety of different Level-*k* heuristics, or somehow managed to coordinate on the Nash equilibrium (NE)—it is easy to verify that in this example there exists a non-monotonic relationship between the level of sophistication and payoffs. Of course, this is not necessarily so for other games. If players 1 and 2 both use the Level-2 heuristic under no time pressure (which entails beliefs that their opponent is Level-1), then they play M-R and their resulting payoffs are 12 and 41, respectively. If under time pressure the players restrict their beliefs about the opponent to be Level-0, then they both play the Level-1 heuristic (actions U-L) resulting in payoffs of 57 and 58. Thus *both* players are *better off* under time pressure. Spiliopoulos et al. (2015) confirm the hypothesis of strategy shift from more to less sophisticated strategies under time pressure in $3\times 3$ normal-form games. Specifically, subjects switched from Nash equilibrium, Level-2 and other more sophisticated decision rules to predominantly using the simple Level-1 heuristic, which ignores the strategic aspects of the game. Since beliefs were not elicited in this study, we do not know whether the driver of the shift was a change in beliefs—we believe this question to be an interesting one that deserves further attention.

**Table 5:** An example of strategy shift—Game #9 from Costa-Gomes and Weizsäcker (2008)

		*Player 2*		
		L	C	R
*Player 1*	U	57, 58$^{L1}$	46, 34$^{L3}$	74, 70$^{L4= NE }$
	M	89, 32	31, 83	12, 41$^{L2}$
	D	41, 94	16, 37	53, 23

Superscripts denote the outcomes of both players using a Level-k heuristic (abbreviated as Lk) or the Nash equilibrium (NE)

#### Criteria shift

This refers to a change in the level of the decision criterion rather than a change in decision processes or heuristics, e.g. Newell and Lee (2011). Recall that in sequential-sampling models, a decision is made once the evidence in favor of an option reaches the threshold value. Consequently, lowering this value leads to a faster response but typically increases the probability of decision errors and vice-versa.

#### Iteration reduction

Apart from hierarchical beliefs in strategic DM, which rely on iterated computations, there exist other non-belief based strategies that require iterated reasoning. Similarly, we hypothesize that under time pressure fewer iterations will be performed by players. Examples include iterated deletion of dominated strategies and backward or forward induction (or lookahead) strategies.

## Footnotes

1 Note, the term *incentivized* is typically used to refer to choice and performance-related payments; since here we use it in the context of RT, the term signifies whether there is a benefit to responding faster.

2 Level-1 reasoning assumes that an opponent randomizes with equal probability over his/her action space and best responds to this assumption. Note, that it essentially ignores the strategic qualities of the game.

3 See Table 2 in our working paper (Spiliopoulos and Ortmann 2016) for a timeline of studies.

4 See for example the vast literature comparing Expected Utility Theory, Cumulative Prospect Theory and other alternatives for decision making under risk.

5 We used the following search strings: + “decision making” + “decision time”, + “decision making” + “response time”, +decision + “response time”, +game + “response time”, + “game theory” + “response time”, “game theory” + “decision time”.

6 In *decisions from description*, subjects are given both the value and the associated probabilities for each outcome. In *decisions from experience*, possible outcomes and their associated probabilities of occurring must be learned through sampling, that is, through repeated draws with replacement from payoff distributions unknown to the decision maker (Barron and Erev 2003; Erev and Roth 2014; Hertwig et al. 2004; Hertwig and Erev 2009).

7 For example, many heuristics can be broken down into three basic building blocks that specify the performed operations: search rules, stopping rules and decision rules.

8 However, RT in repeated games may decrease *between* decisions, i.e., across rounds of play, if the player has an epiphany/eureka moment that allows her to solve future rounds more quickly (Dufwenberg et al. 2010; McKinney and Huyck 2013; Schotter and Trevino 2014b).

9 We thank an anonymous referee for this suggestion.

10 For example, consider experiments run in the laboratory versus Amazon Mechanical Turk; comparing the RT of subjects across these would be particularly insightful in determining whether an equivalent amount of effort is put into the tasks.

11 Reimers and Stewart (2014) find that the average error is approximately 25 ms across different hardware and software (in this cases different web-browsers). de Leeuw and Motz (2015) find similar differences between the Matlab Psychophysics Toolbox and Javascript. Hilbig (2016) compares online-based experiments to those in the lab using E-Prime and Javascript/HTML and concludes that all methods could reliably detect differences in RT of the order of 200 ms.

12 The hierarchical approach assumes that individual-specific parameters are randomly drawn from a distribution whose hyper-parameters must be estimated. For example, two hyper-parameters are required for a normally distributed parameter instead of *n* individual-specific parameters, where *n* is the number of individuals.
